# Strength in diversity: unlocking the full potential of engineered living materials with multistrain collaboration

**DOI:** 10.1093/femsre/fuaf055

**Published:** 2025-11-06

**Authors:** Hannelore Wilssens, Lien De Wannemaeker, Marjan De Mey

**Affiliations:** Centre for Synthetic Biology (CSB), Ghent University, Coupure Links 653, B-9000 Ghent, Belgium; Centre for Synthetic Biology (CSB), Ghent University, Coupure Links 653, B-9000 Ghent, Belgium; Centre for Synthetic Biology (CSB), Ghent University, Coupure Links 653, B-9000 Ghent, Belgium

**Keywords:** engineered living materials, microbial consortia, division of labour, bio-ELM, synthetic biology, sustainable materials

## Abstract

In the innovative field of engineered living materials (ELMs) microbiology and material sciences meet. These materials incorporate living organisms, such as bacteria, fungi, plants, or algae, to enable unique functions like self-assembly, actuation, and dynamic interaction. By utilizing (micro)biological systems in material design, ELMs promise to transform industries including healthcare, construction, and agriculture. In the early phase of ELM technology development, researchers implemented a single living strain in an already established user material. However, the complexity and potential of these materials is limited by the abilities of this single strain. Even though synthetic biology brings the opportunity to add a range of nonnative bioactivities to these cells and thus the material, the increasing metabolic burden upon implementation of multiple nonnative pathways limits the capacity of a single strain. Furthermore, higher organisms and nonstandard hosts are often desired in material settings for their native physical or metabolic advantages. However these are not always straightforward to further engineer. Thus, the use of multiple, specialized strains broadens the functionalities and thus the applicability of ELMs. Multistrain ELMs are a brand-new technology, with many promising applications.

## Teaser

This review describes a modular and rational design framework for engineered living materials, enabled by the functional diversity of multistrain microbial consortia, to create targeted, responsive, and fully biological materials.

## Exploring engineered living materials at the intersection of biology and materials science

Engineered living materials (ELMs) represent a groundbreaking innovation at the intersection of biology and material sciences. They combine two of the most prominent trends in the current material industry: (i) the development of smart and tuneable materials (Khan et al. 2020, Sobczyk et al. 2022) and (ii) the trend towards nature-inspired materials (Karimah et al. 2021, Ashby 2022). The term ELM was first used for a program run by Defense Advanced Research Projects Agency (DARPA) in 2016 (DARPA.mil 2016), later in a first publication by Nguyen (2017) in 2017 and has been booming ever since. In 2022, a clear perspective article was published on the first use, evolution and classification of the term ‘engineered living material’ (Lantada et al. 2022). ELMs are defined as materials containing or consisting of living cells that are engineered, either genetically or mechanically, to add or modulate a specific material characteristic or functionality. These remarkable materials are designed to blur the traditional boundaries between the living and nonliving worlds and harness the power of living organisms, such as bacteria, fungi, plants, or algae, to self-assemble, actuate, and interact. These cells being alive differentiates an ELM from a common biological material and holds the possibility to include a desired bioactivity in the material. Moreover, the recent advances in synthetic biology open up a broad range of bioactivities that can be engineered in living cells and allow for strict regulation of these features (Lee et al. 2018a). By exploiting biological systems in a materials setting, ELMs can revolutionize multiple industries, as diverse as healthcare, construction, or agriculture (Tang et al. 2021). In the last decade, a major part of ELM development was situated in the healthcare industry, building on the profound research on biomaterials. This led to the development of probiotic or drug releasing materials (Sankaran et al. 2019, Sabio et al. 2021), generation of 3D mammalian tissues, and medical detection tools (Mimee et al. 2018, Usai et al. 2023). A prominent example is an ingestible monitoring device combining engineered biosensor bacteria and data-processing microelectronics for management and diagnostics of gastrointestinal diseases, such as gastrointestinal bleeding in swine (Mimee et al. 2018). The device contains engineered bacterial cells that luminesce in the presence of certain small molecules after which this bioluminescence is detected, amplified, and transmitted wirelessly by the electronic system.

ELMs are classified in two groups: biological ELMs (bio-ELMs) and hybrid ELMs. Bio-ELMs entirely exist of biological cells and/or components produced by those cells, while hybrid ELMs are a combination of biological cells and synthetic compounds, like synthetic polymers, metals, or ceramics (Lantada et al. 2022). Currently, most ELMs are hybrid biomaterials consisting of a synthetic scaffold and a living organism, which provides a specific functionality to the material. Researchers have been further exploring this field for a couple of years by combining multiple organisms on these scaffolds, improving the features and possibilities of these materials even more (Nguyen et al. 2018). In the last couple of years however, more and more bio-ELMs are being developed (Huang et al. 2022a, Schenck et al. 2015, Gilbert et al. 2021, Rodrigo-Navarro et al. 2021, Vandelook et al. 2021). Not relying on synthetic compounds, bio-ELMs contribute more to the general transition towards a more circular, sustainable, and ecofriendly bio-based economy.

In the early phase of ELM technology development, researchers implemented a single living strain in an already established user material. These can be considered as proofs of concepts for (i) successful use of living cells and organisms in application-ready materials, (ii) broadening the potential of these materials by genetically engineering the organisms and cells, and (iii) introducing the concept of ELMs to the (scientific) community. As these technologies are getting more established and perspectives for more complex ELMs are emerging, interest in multistrain materials is rising. In addition to nature providing multiple examples of the merits of cocultivation, there are the technical aspects guiding scientists in the direction of cocultivation. First of all, desired functionalities in ELMs can often only be established via a multistep process. Engineering a single cell type or organism to perform all the necessary intermediary steps for such a complex functionality can be complicated and inefficient. This can be overcome by including multiple specialized organisms, each performing a smaller, simple biological task. Each desired characteristic can be established by a specialized organism in a multistrain ELM. Second, cocultivating these complementary organisms leads to a consortium that performs the complex functionality without creating additional burden for the single organisms (Nguyen et al. 2018). This review will focus mainly on multistrain bio-ELMs, where at least one of the cell types remains viable during the use phase of the material. This includes ELMs containing (leftovers from) a synthetic compound that is utilized by organisms to form the material, e.g. silica to induce biomineralization or substrate for filamentous fungi and excludes ELMs that are incapacitated after the production phase or solely exist of components produced by living cells. More specific, the review sheds light on the advances in the use of cocultivation for ELM development. Therefore, a classification of possible functions of living cells in bio-ELMs in general is presented, followed by highlighting the possibilities and task division of organisms in a multistrain bio-ELM with examples drawn from diverse disciplines. This classification can be used by researchers to create new ELMs in a modular way by rationally selecting and combining organisms depending on the desired characteristics for the material. Lastly, the main bottlenecks that currently prevent the development of industrially relevant multistrain ELMs are discussed. Identifying the state-of-the-art, progress and future perspectives in the field of bio-ELMs, helps to identify the bottlenecks to be tackled in order to enhance control and predictability in the design and development of multistrain biomaterials. Altogether, this review article illustrates the revolutionary impact of cocultivation on the development of sustainable materials and shaping a more sustainable future for current and future generations.

## Exploring the key roles of microorganisms in ELMs

A defining strength of ELMs is their ability to harness dynamic biological activities. Hence, the vast amount of bioactivities that living cells capacitate creates a wide variety of functionalities and consequent end applications of ELMs. This includes natural bioactivities but is continuously expanding with the current development in synthetic biology. To understand the design of more complex multistrain bio-ELMs, a classification of the possible purposes a viable organism can have within ELMs is presented first.

**Figure 1.**
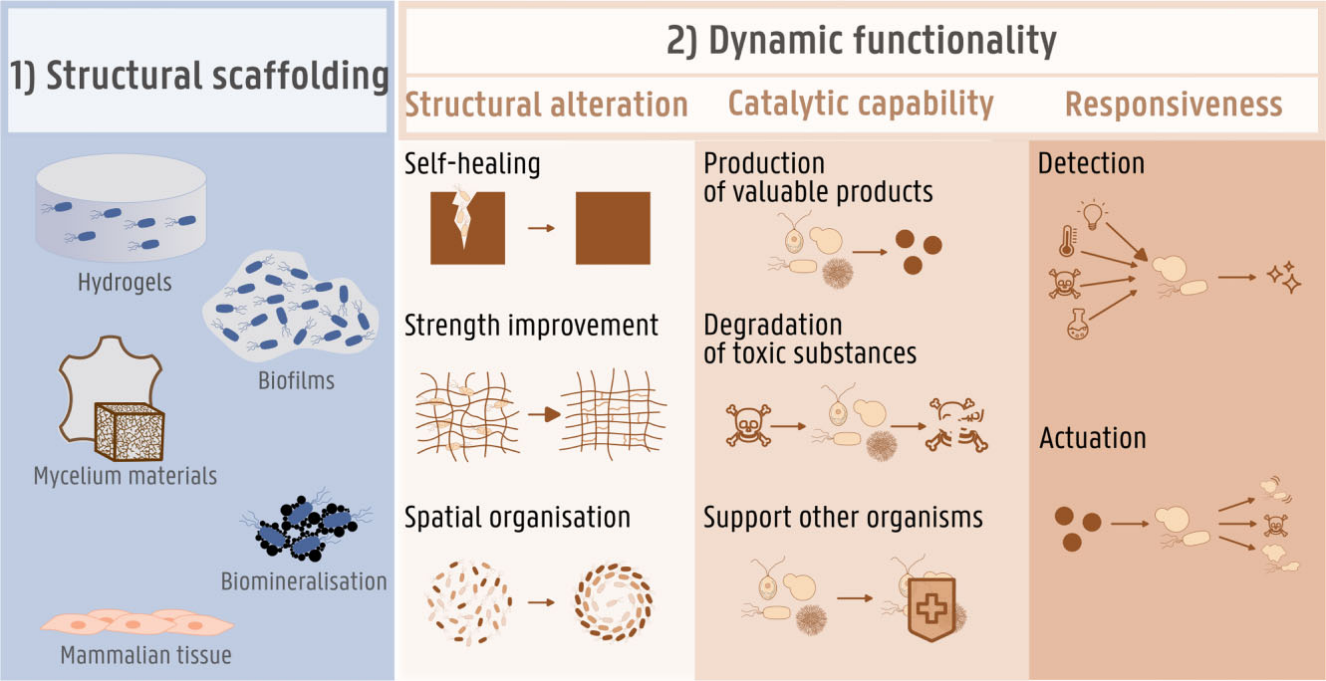
Schematic categorization of living organisms in ELMs. The two main aspects within an ELM are: (1) the structural scaffold and (2) the dynamic functionality. The function of all living organisms in currently developed ELMs can be assigned to one of these subcategories.

When considering the added value of living cells in materials, two main categories can be distinguished: structural scaffolding and dynamic functionality (Fig. 1). In the former category (structural scaffolding; Fig. 1, panel 1), the material scaffold is either produced by or consisting of the living cells. In the latter category (Fig. 1, panel 2), the living cells add a dynamic functionality, allowing them to swiftly respond to external changes and hence gives ELMs a major advantage over nonliving materials. Three aspects can be defined within this dynamic functionality: (i) structural alterations, (ii) catalytic capability, and (iii) responsiveness. These dynamics can be implemented either in the production or use phase of the material. Cells yielding dynamic functionalities often need support from a synthetic or biological scaffold. This usually results in multistrain bio-ELMs that combine different strains: one provides structural scaffolding (category 1), while another offers dynamic functionality (category 2). The following sections will further illustrate and discuss these categories and their underlying aspects.

### Structural scaffolding

In order for a material to be implemented in practice, it should have application appropriate structural characteristics in terms of hardness, strength, elasticity, and so on. Moreover, society is not eager to accept materials with a different feel or appearance than what they are used to (Polyportis et al. 2022, van den Broek et al. 2022, Bonenberg et al. 2023). Therefore, the structural scaffold in ELMs often consists of a synthetic material to gain known and desired physical material properties, resulting in a hybrid living material. Yet, more research is going into (living) biological scaffolds with commercially interesting physical properties. This approach is among others on the rise in the soft robotics, biotechnological, and medical sector (Wang et al. 2024b, Appiah et al. 2019, Hernández-Arriaga et al. 2022).

The most prominent approach for biological living structural scaffolds is biopolymeric soft materials produced by microorganisms (cellulose, DNA…). The main advantage of these biologically produced polymers is that their physical and chemical properties can be tailored by bioengineering (Hernández-Arriaga et al. 2022, Gaspers et al. 2025). Mezzina et al. (2021) illustrated this by altering the Young’s modulus, thermal behaviour and crystallinity of *Pseudomonas putida*-produced polyhydroxyalkanoates (PHAs) by metabolic engineering and feeding approaches. The different kinds of biopolymers produced by bacterial producers and their major field of application has been summarized by Hernández-Arriaga et al. (2022). In particular, bacterial cellulose has shown to trigger interest in different research fields. The pathway and regulation mechanism for this extracellular biopolymer has been characterized in, but is not limited to, *Komagataeibacter* (*K. xylinus*) (Ryngajłło et al. 2020) and results in a porous hydrogel, perfect for use in soft bioelectronics (Huang et al. 2022a, Appiah et al. 2019, Wang et al. 2021) or 3D bioprinting (Hammad et al. 2025, Hunsberger et al. 2025). Moreover, the biocompatibility and porosity make these bacterially produced materials perfect scaffolds for immobilization of living cells, enabling cocultivation for further enhancement of the material characteristics and abilities (Gilbert et al. 2021).

In addition to being produced by living cells, the structural scaffold in ELMs can also be composed of them. This is for example the case in medical tissue engineering, where the cells form a graft to replace or support the growth of mammalian tissues. Yaguchi et al. (2016) fabricated an autologous mucosal graft to be implanted in the middle ear after surgery on middle ear infections. Alongside medical tissue engineering, the packaging industry has growing interest in biological materials and in particular mycelium-based materials. Mycelium is becoming more popular as a sustainable material component, occurring as foam-like materials, compact composite, or leather-like materials (Jones et al. 2020, Vandelook et al. 2021, Raman et al. 2022). They are already being produced and implemented on an industrial scale, by companies like IKEA or DELL as biodegradable packaging material (Cumbers 2023). Although, in current industrial applications, the mycelium is killed off after the growth phase, missing out on the opportunities living materials bring, like self-healing or continuous valorization of organic waste streams (Wang et al. 2024a, Elsacker et al. 2023).

### Dynamic functionalities

In addition to providing a static scaffold, living cells have the ability to (i) dynamically alter the structure of the material, (ii) perform a catalytic reaction, or (iii) sense and react in a predictable manner (Fig. 1, panel 2). These dynamic functionalities can emerge without any human or robotic intervention because of the (naturally) programmed mechanisms in a living cell.

#### Structural alterations

Research on dynamic structures in ELMs can be broadly categorized into three main areas: (i) self-healing, (ii) strength enhancement, for example through cross-linking, and (iii) spatial organization of the material, as illustrated in Fig. 1. The most well-known example of dynamic structural alterations by living cells in a practical material setting is self-healing concrete, where bacteria are incorporated in the concrete. At the surface of the concrete or in the presence of a crack, moisture or water activates the metabolism of the bacteria and biomineralization takes place. This additionally decreases gas and water permeability, which dynamically increases the durability of the material (De Muynck et al. 2008, Akindahunsi et al. 2021). This hybrid ELM served as inspiration for multiple multistrain bio-ELMs, where the concrete is replaced by a biological scaffold (Birnbaum et al. 2021, Yu et al. 2021, Viles et al. 2025). Microbially induced calcium precipitation (MICP) enhances the strength of these hydrogels or mycelium scaffolds (Heveran et al. 2020, Yu et al. 2021). Additionally, considering research on MICP in nonbiological scaffolds, durability of the biological materials could be enhanced by lowering erosion, suppression of dust formation, improving thermal conductivity, or local self-healing (Zhang et al. 2023a). Research on spatial organization in ELMs is currently mainly conducted on a fundamental level, but once applied it could allow for autonomous hierarchical growth of the biological material of tissue development or the development of an ‘assembly line’-like material consisting of specialized subunits to cover complex metabolic pathways. Dynamic alterations of a material structure are important for applications that require continuous growth of the material, *in situ* spatial organization, or locally induced strengthening.

#### Catalytic capability

A second dynamic advantage living cells can bring to a material is the catalytic capability. The three main catalytic functionalities living cells can bring to ELMs are (i) production of valuable products, (ii) degradation of toxic substances, and (iii) the support of other organisms (Fig. 1). (Micro)organisms produce a wide range of enzymes that serve two main functions. On one hand, these enzymes break down specific compounds in the environment, providing nutrients and energy. On the other hand, they help build essential components that support cell growth and protect the organisms from competitors and unfavourable conditions. Their ability to produce valuable compounds has been harnessed extensively in the field of industrial biotechnology, where antibiotics, vitamins, chemicals, sugars, and innumerable more compounds are produced in contained fermenters (Buchholz and Collins 2013, Fedorenko et al. 2015, Averianova et al. 2020, Francois et al. 2020, Bilal et al. 2021). The successfully exploitation of these microorganisms established a platform for a bio-based economy and therefore boosted further research in metabolic engineering and synthetic biology (Clarke and Kitney 2020). Applying the knowledge gained in industrial biotechnology to ELMs would yield for example antibiotic producing grafts for wound healing or biofouling-resistant filter membranes (Gerber et al. 2012, Wood et al. 2016). The opposite ability, to break down compounds, has a clear application in bioremediation. When soil or water is contaminated with toxic compounds or heavy metals, (engineered) organisms break down the toxic compound or sequester the heavy metals (Tay et al. 2017, Zhao et al. 2023). Another catalytic function that can be utilized in ELMs is the creation of a beneficial microenvironment with specific levels of oxygen, CO_2_ or other nutrients in order for other organisms to thrive (Schenck et al. 2015, Yen et al. 2015). This is especially interesting in multistrain ELMs. However, engineering any of those three aspects creates a metabolic burden, resulting from engineering extensive synthetic pathways into the organisms. This can be avoided by dividing the pathway and thus the burden over multiple differently engineered organisms as will be clarified later.

#### Responsiveness

The last aspect of dynamic functionalities in ELMs is responsiveness (Fig. 1, panel 2). Microorganisms have biological mechanisms to precisely monitor changing extra- and intracellular concentrations of specific molecules, metal ions, CO_2_, O_2_, pH, light, and temperature (Kiel et al. 2010, Gui et al. 2017). These mechanisms are essential to enable them to adapt their metabolisms in order to maximize metabolic resource management, to steer away from dangerous situations, or to explore and exploit new niches. In contrast to structural alterations and catalytic capability, this ability is often strictly regulated through a complex metabolic pathway with multiple steps that have been fine-tuned over millions of years to result in an appropriate reaction. Since the emergence of synthetic biology, researchers have exploited the natural ability of organisms to sense endo- or exogenous small molecules, ions, and changes in physical parameters (De Baets et al. 2024). Biosensing cells are created by introducing a genetic circuit that combines this cell’s inherent monitoring system with and output signal of choice, such as fluorescence (De Paepe et al. 2017). Within a specific range, the output level can be linked with the input concentration, which can be extremely useful for *in vivo* medical or environmental monitoring applications (Bahadr and Sezgintürk 2015, Demeester et al. 2023, Yunus et al. 2023). Apart from detecting a wide variety of input signals, living cells are able to actuate as a reaction on these inputs. Actuation can exist of change in cell morphology, cell movement, switch of metabolic pathway, cell death, or cell revival (Park et al. 2016, González et al. 2020, Rivera-Tarazona et al. 2020, Chiang et al. 2022).

## Leveraging organism diversity for expanding capabilities in biological ELMs

The classification mentioned above and depicted in Fig. 1 concerns the value of any single type of organism, viable in the production and/or use phase of ELMs. As technologies are being developed increasingly for each of these subcategories, a next milestone in the development of living materials is to combine two or more subcategories in order to enhance the possibilities of these materials even more. This principle has been applied by multiple prototype ELMs. The next sections will focus solely on biological ELMs (bio-ELMs) including two or more different cell types of which at least one stays viable in the use phase. ELMs that include synthetic compounds used by the organism to form a material are included in the selection as long as the synthetic compounds do not form a material in itself; they are located in between bio- and hybrid ELMs. An overview of prototype bio-ELMs that integrate functional subcategories through the combination of multiple strains is provided in Table 1.

**Table 1.**
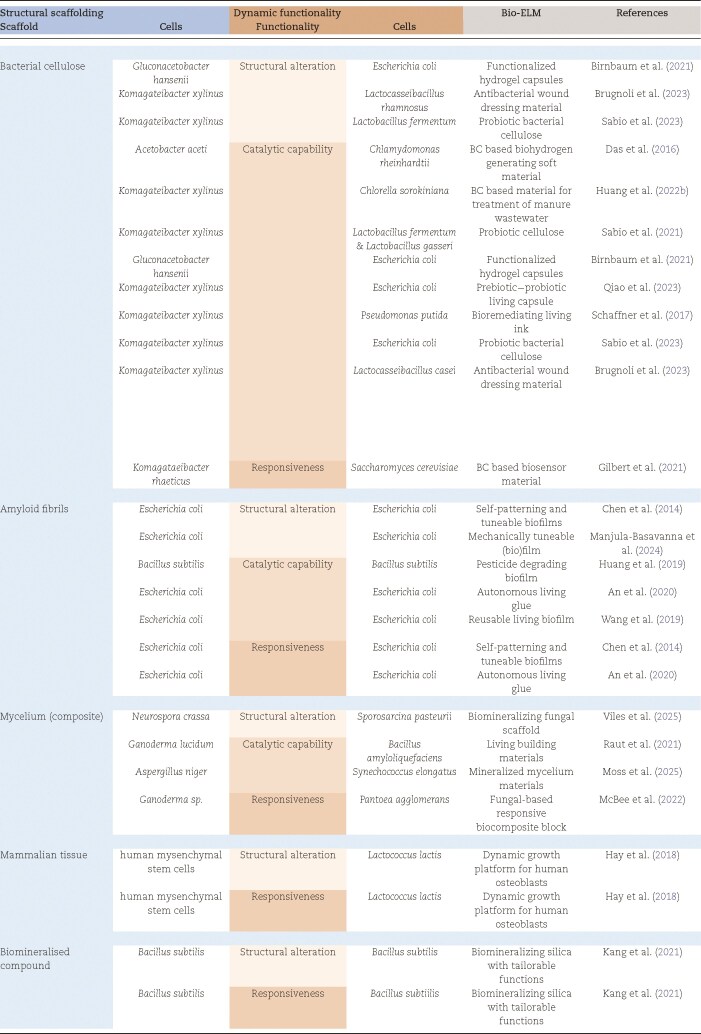
Application of the classification of functionalities of organisms to multistrain ELMs described in literature. ELM = engineered living material.

### Combining biological scaffolds with dynamic capabilities

**Figure 2.**
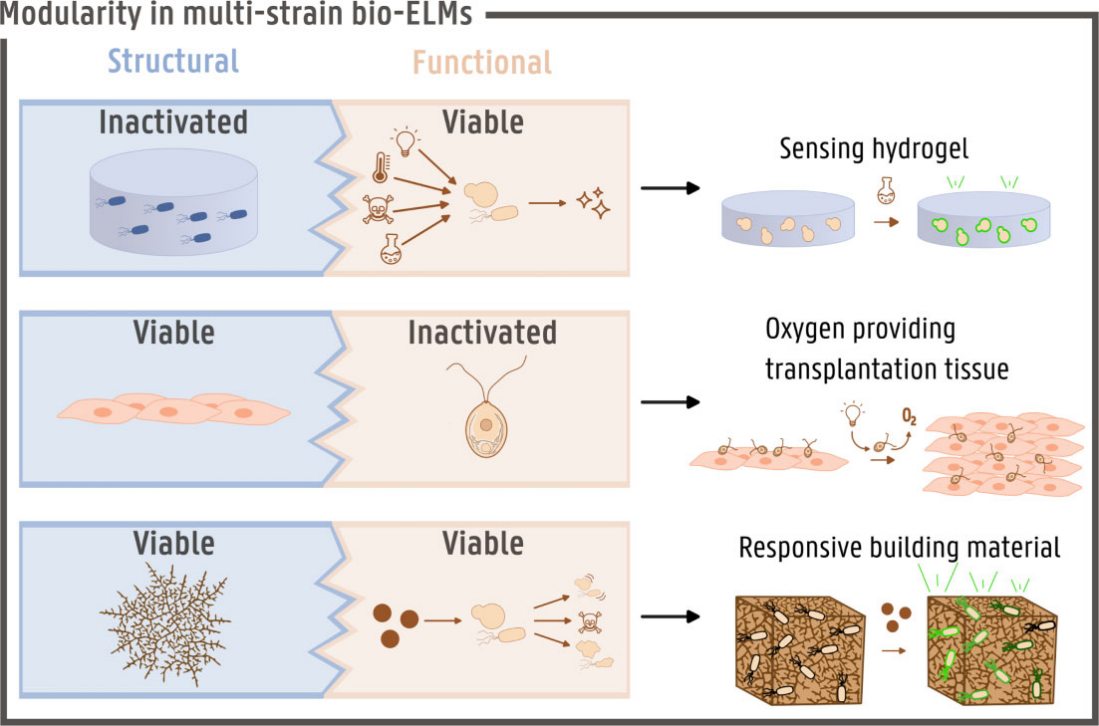
Examples of modularity in multistrain biological ELMs described in literature, displaying the possibilities of combining viable and inactivated biological compounds. Every organism has a distinct role (left: structural, right: functional), leading to unique combinations and applications of the materials. Top: a bacterially produced hydrogel, in which the bacteria have been inactivated, is combined with genetically engineered yeast or bacteria to create a biosensing hydrogel. Middle: algae supply oxygen during the developmental phase of engineered tissue and are subsequently inactivated prior to transplantation. Bottom: a living multistrain material is formed by maintaining both structural (e.g. mycelium) and functional (e.g. bacteria or yeast) components in a viable state, enabling self-growth and environmental responsiveness. Bio-ELMs = biological engineered living materials.

As shown in Table 1, most multistrain bio-ELMs combine one organism providing structural scaffolding with another that delivers dynamic functionality. This approach is unsurprising in bio-ELM design, as structural scaffolding is fundamental to material formation, while dynamic functionality is a defining characteristic of living materials. As these two categories (panels 1 and 2 in Fig. 1) require very distinct properties from the organism representing it, often a different species is selected for each of these categories (Fig. 2) (Das et al. 2016, Hay et al. 2018, Birnbaum et al. 2021, Huang et al. 2022b, Gilbert et al. 2021, Kang et al. 2021, Răut et al. 2021, Sabio et al. 2021, McBee et al. 2022, Qiao et al. 2023, Moss et al. 2025). Additionally, in the majority of the cases one of the organisms is killed off after fulfilling its role. Hence, only one of these partners stays viable in the use phase of the material; this can be any of the partners. For example in the research of Gilbert et al. (2021), bacteria produce a cellulose scaffold that host engineered baker’s yeast that is able to sense a chemical activator and respond to it by expressing fluorescent reporter proteins. In this case, the functional partner, yielding responsiveness, stays alive in the use phase and the partner providing the materials structure is killed off before use. The reverse combination, where the scaffolding partner stays alive and the dynamic partner is the temporary support, has been illustrated by Haraguchi et al. (2017). A scaffold of mammalian cells was created to guide the growth of new cells in case of tissue injuries. The mammalian cells were supported by photosynthetic (micro)algae that provided these with oxygen, avoiding hypoxia and improving tissue formation for wound healing and that are heat inactivated by the host body upon transplantation. Moreover, a similar prototype bio-ELM with both functional and structural partner remaining viable has been successfully demonstrated by transplanting scaffolds in mouse tissue and zebrafish containing living mammalian and algal cells without inflammatory response (Schenck et al. 2015). Another example of a completely viable bio-ELM is the regenerative fungal–bacterial material that combines a regenerative mycelium structure with bacteria that are able to react on the presence of a ‘sender’ strain (McBee et al. 2022). These examples thus nicely illustrate the fact that when combining structural scaffolding and dynamic functionality in a bio-ELM, different species—in this case even different domains—are selected. These species survive independent from each other, avoiding the complexity of biocompatibility and biological warfare. On the other hand, multistrain ELMs, combining functionalities within one category, often consist of one strain that is differently engineered in order to yield distinct functionalities (Chen et al. 2014, Hay et al. 2018, Huang et al. 2019, An et al. 2020, Kang et al. 2021, Jin et al. 2022, Manjula-Basavanna et al. 2024, Hammad et al. 2025, Moss et al. 2025). This approach offers advantages like ease of handling, biocompatibility, and similar growth conditions compared to multispecies cocultivations, but requires model organisms with an extensive synthetic biology toolbox, like *Escherichia coli* or *Saccharomyces cerevisiae*. The model organisms used today, however, sometimes stand far from the ideal organisms for the application envisioned (De Wannemaeker et al. 2022). Archaea, for example, are naturally hardened against extreme environmental conditions and could thus be perfect candidates for ELMs destined for outdoor use. However, these organisms have none or less tools available for genetic engineering and are thus limited in use to their natural capabilities (Pfeifer et al. 2021, Aparici-Carratalá et al. 2023). This indicates the need for research on synthetic biology tools for nonmodel organisms, enabling the coupling of novel capabilities, introduced by synthetic biology, with the natural benefits of these extraordinary organisms (Calero and Nikel 2019). The future in this regard looks very promising as there is a trend in synthetic biology towards modularity and cross-species engineering (De Wannemaeker et al. 2022, Martínez-García et al. 2023). This way, bio-ELMs can be built in a modular manner, selecting perfect candidates for every desired characteristic or functionality in the material. However, as will be discussed later in the review, more fundamental knowledge is necessary about the behaviour of living organisms in a material setting and the interaction between multiple species for this modularity to be established and predictable.

### Dominant trends in multistrain bio-ELMs

In first generation multistrain bio-ELMs, two major trends can be detected in the design of the materials, being (i) that the structure of the materials is predominantly consisting of hydrogels and (ii) catalytic capabilities are in the majority of the cases the dynamic functionality of choice.

#### Material structures dominated by hydrogels

**Figure 3.**
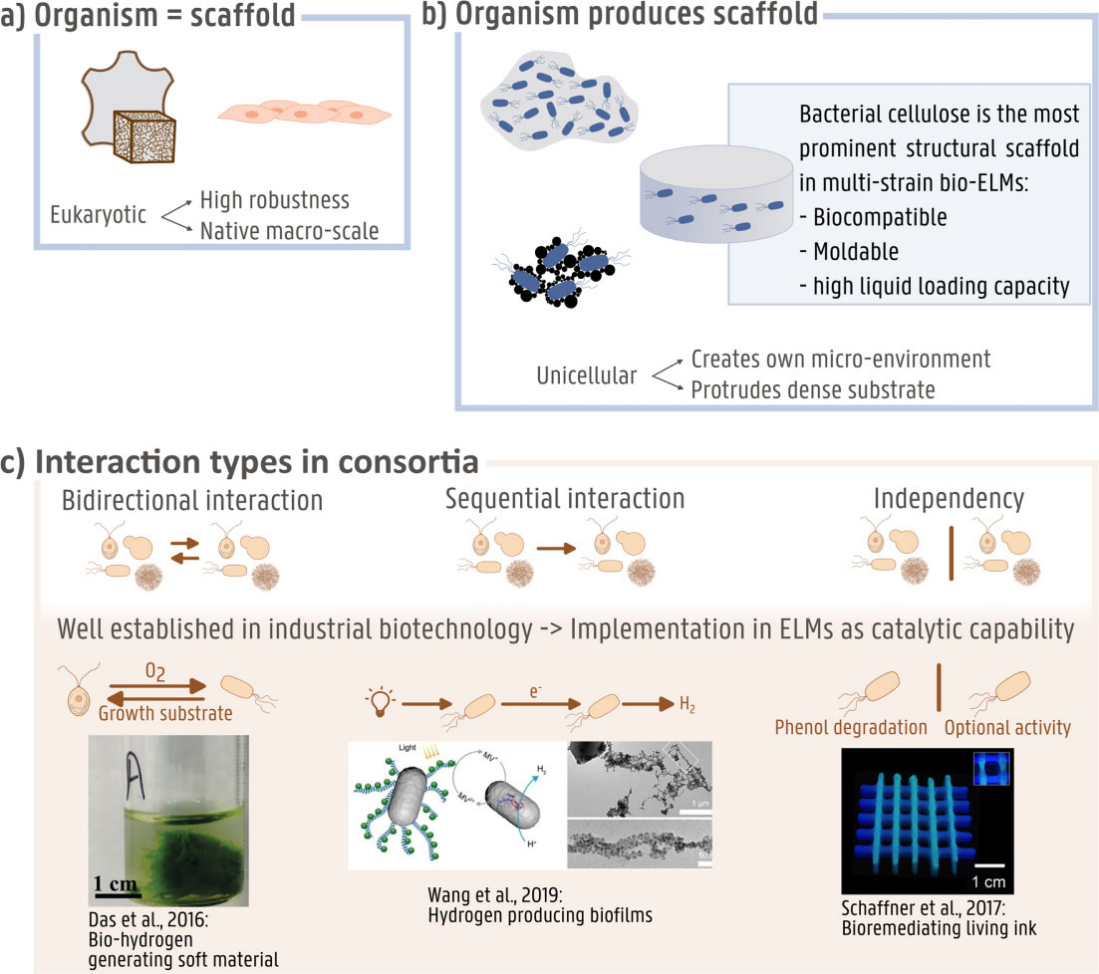
The already developed multistrain ELMs show clear trends regarding the deployment of organisms, depicted in the different panels. (A) In materials where the structure is made of cells, eukaryotic or multicellular organisms are mostly harnessed due to their natural robustness and macroscale. Examples of biological scaffolds given here are mycelium and eukaryotic cell tissue. (B) Unicellular organisms are implemented for producing a structural scaffold due to their ability to protrude dense substrates and creation of a hospitable microenvironment. Overall, the structure of the material in multistrain ELMs is dominated by bacterially produced cellulose. The biocompatibility, moldability, and high liquid loading capacity make it the perfect basis for hosting microbial partners and producing a durable, widely applicable and harmless material. (C) The dynamic functionality in an ELM can or cannot be introduced by an organism interacting with other organisms in the material. Microbial consortium interactions are being harnessed in industrial biotechnology as a strategy for complex catalytic processes. These principles can be transferred to ELM technologies. Each type of interaction is illustrated with an example from literature. Left: algae encapsulated in a bacterially produced hydrogel provide oxygen for the bacteria and benefit from the growth substrate provided by the bacteria in a bidirectional interaction (Das et al. 2016). Middle: sequential interaction between differently engineered bacterial strains in a biofilm by division of labour. The different strains create a pathway to produce hydrogen from light (Wang et al. 2019). Right: two independently acting bacterial strains with different capabilities combined in one material by printing a bacteria–hydrogel mixture in a pattern (Schaffner et al. 2017). (Bio-)ELM = (biological) engineered living material.

Within an ELM, the structural scaffold is the backbone that resembles the basis for all nonbiological properties of the material, like tensile strength, elasticity, and shape. In multistrain bio-ELMs, these scaffolds additionally have to be appropriate to retain and host other living organisms (Vandelook et al. 2021, Jin et al. 2022). This combination of biocompatibility and macro-scale consistency can be established by either cells, inherently forming macro-scale structures, like mammalian cells or filamentous fungi, or by biopolymeric materials like biofilms or hydrogels (Fig. 3a). Research shows that multicellular organisms and other eukaryotes have high levels of robustness leading to better fitness in a constant environment and less variation in abruptly changing environments than the highly adaptable unicellular organisms (Jiang et al. 2023). Since the lifespan of an ELM is rather low in evolutionary terms, the high robustness of multicellular and other eukaryotic organisms is desired. Moreover, a stable material structure is preferred over one that adapts to the smallest environmental perturbation. To this regard, filamentous fungi, are booming in the sector of biocomposites as isolation, packaging, or building materials; proving their use as a structural scaffold (Cerimi et al. 2019, Jones et al. 2020, Elsacker et al. 2023, Moss et al. 2025). McBee et al. (2022) described a dormant fungal structural scaffold, holding engineered bacteria, that was stable, could be resurrected after a year in ambient conditions and had no loss in material strength after four full generations of regeneration. On the other hand, even though unicellular organisms are less robust, they are able to produce compounds to create a stable microenvironment for themselves. Biofilms, produced by prokaryotes for example, can provide sufficient protection against different temperatures, pH levels, salinity, radiation, and even antibiotics to serve as a scaffold to retain partner organisms (Williams et al. 2009, de Carvalho 2017, Cepas et al. 2019, Tran et al. 2024). Therefore, biofilms have been applied as bioink or bioremediating films (Tay et al. 2017, Huang et al. 2019). A major benefit of scaffolds that are produced by microorganisms rather than being the organism itself is thus that these scaffolds are not metabolically variable depending on external factors. Additionally, unicellular microorganisms can easily protrude dense substrates, which is beneficial for biomineralization purposes (Kang et al. 2021, Viles et al. 2025). The most prominent microbially produced structural scaffold used in multistrain bio-ELMs, however, is bacterial cellulose (BC). These gel-like scaffolds are nontoxic, biocompatible, mouldable, and have a high liquid loading capacity and good mechanical properties, making this material the perfect renewable biomaterial and environment for cocultivated organisms (Ghozali et al. 2021). In recent research, microbial partners holding the dynamic functionality were entrapped within this cellulosic network and this way protected from external factors like dehydration and antimicrobials (Das et al. 2016, Birnbaum et al. 2021, Huang et al. 2022b, Gilbert et al. 2021, Sabio et al. 2021, Qiao et al. 2023). The class of organisms producing the structural scaffold is thus dominated by prokaryotes and more specifically the bacterial domain (Fig. 3b)

#### Catalytic capability as main focus in dynamic functionalities

The field of ELMs aims to implement the dynamics of biological systems into a material setting. Many biological dynamic systems are formed by consortia rather than single cell types, where cells take advantages of each other’s unique abilities. These abilities can range from providing nutrients to providing a physical shelter or sharing the burden for disintegrating toxic compounds (Zhang et al. 2016, Rafieenia et al. 2022). This concept, called division of labour, has already been applied in fermentation processes, such as anaerobic digestion, to lower the metabolic burden on single cell types and improve production titres (Zhang et al. 2023b). Division of labour can artificially be implemented through spatial segregation, metabolic engineering or feeding strategies (Giri et al. 2019). Despite the great potential, synthetic microbial consortia are mostly limited to academic research due to challenges of stability and controllability (Ben Said and Or 2017, Rafieenia et al. 2022).

Although the field of ELMs has not developed as far as including complex synthetic consortia in the material, first generation multistrain bio-ELMs do combine a limited amount of strains with a dynamic functionality, expanding and improving the possibilities of bio-ELMs (Rapp et al. 2020, Wang et al. 2022, 2024b). These strains often have a direct influence on the state or function of one another (Fig. 3c). For instance, self-patterning and dynamic spatial organization require close interaction of multiple organisms and has been observed and studied in well-defined bacterial consortia (Tecon and Or 2017). This has been mimicked by engineering the same strain in different ways that are compatible and, when combined, form an autonomous or externally controlled patterning system that can be applied to bio-ELMs (Chen et al. 2014, Toda et al. 2018). In this case, there is a bidirectional dynamic interaction between the organisms, while in other cases, like bacterial cells that induce cell differentiation and enhance attachment of human mesenchymal cells, one of the organisms is purely in service of the other (Hay et al. 2018, Moss et al. 2025). Moreover, not all multistrain bio-ELMs include interaction between organisms. Dynamic properties like strength improvement or self-healing are established through interaction of the dynamic organism with molecules, either produced *in situ* or added externally (Birnbaum et al. 2021, Kang et al. 2021). Furthermore, the dynamic reaction of organisms on external signals is mostly independent of other organisms in the ELM (Chen et al. 2014, Hay et al. 2018, Gilbert et al. 2021, McBee et al. 2022).

Although, the examples above illustrate the value of dynamics in bio-ELMs through the addition of structural alterations or responsiveness, most of the current developed bio-ELMs implement a purely catalytic capability as dynamic functionality. This is likely because the genetic engineering of microorganisms for catalytic processes is highly developed in industrial biotechnology, making those organisms easily implementable as proof of concept in material settings (Intasian et al. 2021). This applies to both interactive dynamics between organisms [bidirectional (Das et al. 2016) or sequential (Hay et al. 2018, Huang et al. 2019, Wang et al. 2019, An et al. 2020, 2022b)] as catalysis moderated by a single cell type (Schaffner et al. 2017, Birnbaum et al. 2021, Kang et al. 2021, Răut et al. 2021, Sabio et al. 2021, 2023, Brugnoli et al. 2023, Qiao et al. 2023). For example, a cooperative interaction between organisms has been engineered over different species; photosynthesizing microalgae were grown with a BC producer in order to provide the latter with oxygen and allow for production of a cellulose network, not only at the air–liquid surface, but deeper in the medium, resulting in a 3D soft material. The BC gel immobilizes microalgae, which are capable of producing biohydrogen, a process shown to be more efficient when the microalgae are immobilized (Das et al. 2016). Catalysis moderated by a sequential interaction or independent cells in bio-ELMs mostly involves the direct or indirect production of a valuable product (Das et al. 2016, Hay et al. 2018, Wang et al. 2019, An et al. 2020, Sabio et al. 2021, 2023, Brugnoli et al. 2023, Qiao et al. 2023) or degradation of pollutants (Schaffner et al. 2017, Huang et al. 2019, Wang et al. 2019, 2022b). Examples are probiotics (Sabio et al. 2021, 2023, Qiao et al. 2023), the display of α-amylase that benefits the industrially relevant process of ethanol production (Birnbaum et al. 2021) or a phenol degrading bioink (Schaffner et al. 2017). The implementation of sequential interaction or independent catalysis relies on the complexity of the compound to be produced or degraded. By displaying these enzymes on the cells or direct production or degradation of products *in situ*, costly purification and immobilization of the involved enzymes can be avoided.

In conclusion, living organisms in bio-ELMs serve a variety of purposes, both interacting with one another and acting individually.

## Current bottlenecks in cocultivated ELMs

Although ELMs show great potential, as discussed in this article, they face several critical challenges that hinder their applicability on the market. One significant bottleneck is social acceptance. There has not been a survey yet on the acceptance of ELMs in society, but research on social acceptance of genetically modified foods, genetic engineering in general, and alternative material usage show that the public concerns about ecological safety, long-term health impacts, and general neophobia often overshadows their perception of benefits of genetically modified organisms (GMOs) (Kiran et al. 2023, Maqsood et al. 2024). However, a study on cultural perception of genetically modified foods showed greater acceptance by younger and more educated populations, suggesting the positive impact of scientific education on the public perception (Maqsood et al. 2024). Moreover, acceptance rates were higher in North America and parts of Asia compared to Europe and Africa. This can be attributed to the stringent regulations and more cautious approach in Europe and Africa. The perception on genetically modified foods in all demographic categories of North America and Asia was more positive and can be linked to those two being the largest genetically modified food markets in the world and thus more exposure of the technology (Kiran et al. 2023, Maqsood et al. 2024). This was confirmed by a study on the social acceptance of genetic engineering technology in general, where education and perceived benefits had a great effect on acceptance (Koralesky et al. 2023). Two studies regarding the use of alternative materials report that there is an overall positive perception of mycelium-based building materials among architecture and interior design students and professional architects. However, the results revealed a double standard: participants were enthusiastic about using the materials in their projects but reluctant to incorporate them into their own homes (Bonenberg et al. 2023, Lewandowska et al. 2024). It can be concluded that social acceptance can be boosted by education on genetic modification in a comprehensive way and by information on (i) the benefits it can bring to society and on (ii) the safety measurements that are taken to guarantee safety for both health and environment.

Biosafety of the engineered materials, thus has to be carefully considered. Since these materials are aimed to be distributed and will consequently be in close contact to humans as well as animals and plants, every organism and genetic modification has to be carefully considered in terms of pathogenicity, toxicity, antibiotic resistance, and environmental risks (Wang and Zhang 2019). Biocontainment is thus crucial to prevent unintended release of engineered organisms into the environment. Multiple containment strategies for engineered organisms exist like physical containment, auxotrophy, prevention of self-replication, or synthetic gene circuits like toxin–antitoxin systems or kill switches (Lee et al. 2018b, De Wannemaeker et al. 2022). An alternative approach, relevant for multistrain ELMs is the use of cooperative consortia as a biocontainment strategy. In this case, the functionality or end product, established by division of labour by two or more strains, is only enabled when the consortium is complete. In case of leakage of one or the other strain, the functionality is not undeliberately activated in the environment. Additionally, double auxotrophy can be engineered in the organisms, creating a bidirectional dependency, inhibiting their separate survival upon leakage (Lee et al. 2018b). However, spontaneous mutagenesis can lead to failing of genetic safeguard systems. To counteract this, chemical and physical containment can be combined, resulting in strict containment of genetically engineered organisms in living materials (Tham 2018). This strategy, however, results in a quick loss of viability in the use phase, limiting the usability in long-term applications, like is necessary in for example architecture applications. Currently, a trade-off thus has to be made between extreme biosafety measures or long-term viability and robustness of the organisms in the material.

While the distribution issues and societal acceptance of multistrain ELMs present significant challenges, these are not the only obstacles hindering their large-scale development and production. The design of a multistrain ELM starts off with the selection of suitable organisms for the envisioned functions of the material. Ensuring the coexistence and thriving of these organisms in a synthetic consortium poses a significant challenge due to the limited understanding of organism interactions and the lack of effective methods for managing synthetic consortia (Duncker et al. 2021). Synthetic consortia, used in current applications, are mainly limited to binary cultures as they are limited by the need for compatible environmental conditions (Ben Said and Or 2017). Although the maintenance of synthetic consortia is often circumvented by sequential culturing of the different strains (Qiao et al. 2023) or using differently engineered strains, originating from the same base strain, leading to similar culturing conditions (Chen et al. 2014, Hay et al. 2018, An et al. 2020, Kang et al. 2021), implementing these organisms in a 3D material adds another layer of complexity. The current techniques for assessing cell density and reading out reporter systems are primarily designed for 2D applications or transparent substrates, limiting their applicability deep within 3D materials without material degradation (Gilbert et al. 2021, McBee et al. 2022). Addressing these technical challenges requires more fundamental research into advanced methods for monitoring, imaging, and culturing microbial consortia and biologically derived materials. Unlike inert systems, these living materials are metabolically active and exhibit dynamic behaviour that varies depending on the applied methodology. As a result, the development of reliable and reproducible techniques is inherently more complex, time-intensive, and costly. Additionally, to date, in case of fully biological ELMs, biological scaffolds have varying mechanical properties, limited scalability, and undesired material properties in comparison to synthetic materials (Wang et al. 2022, Alaneme et al. 2023). These technical barriers, however, are expected to be surmountable as technology and knowledge in cocultivation and ELMs advance.

Once these challenges are addressed, the final and perhaps most critical obstacle remains: regulatory approval. There is currently no specific regulatory framework for ELMs in the EU nor the USA. Currently, ELM-based products are regulated based on legislations for the agri-food or medical sectors, which do not cover the needs for recently developed ELMs, like the unintended *in situ* production of compounds or other dynamic behaviour of the materials. However, both the European Commission as the US government are investing in early-stage research on ELMs with funding programs specifically on ELMs (DARPA.mil 2016, Gerratana Barbara 2018, Heveran et al. 2024). In the EU Horizon Pathfinder challenge on ELMs, for example; the ethical, legal, and social aspects of ELMs are clearly highlighted. Moreover, there is a call for action from ELM researchers to create such frameworks as the current unclarity leads to delays the industrial application development of ELM-based products. Moreover, regulations and standards for such materials could highly benefit the safe and sustainable research within the field. Regulations to be considered are on validation, biosafety, maintenance, and waste management of the material (Ebbesen et al. 2024). Once established, however, considering the complex and time-consuming process involved in gaining approval for GMOs, it is possible that securing regulatory clearance for ELMs may also present similar challenges.

## Conclusion and prospects for multistrain ELMs

By exploiting (micro)biological systems in a materials setting, ELMs can revolutionize multiple industries, as diverse as healthcare, construction, or agriculture. In the early phase of ELM technology development, researchers implemented a single living strain in an already established user material. However, the complexity and potential of these materials is limited by the abilities of this single strain. Thus, the use of multiple, specialized strains broadens the functionalities and therefore the applicability of bio-ELMs. Multistrain bio-ELMs are a brand-new technology, emerging across various areas of the ELM field.

When considering the added value of living cells in ELMs, two main categories can be distinguished: structural scaffolding and dynamic functionality. Every living cell included in a living material so far can be attuned to any of these two categories and their subcategories. Understanding this basic classification gives more insight into the design of more complex multistrain ELMs. Existing literature on already developed, multistrain, fully biological ELMs shows that most combine one organism for structural scaffolding with an organism delivering the dynamic functionality. A more detailed look into these categories shows that the structure of the materials is predominantly consisting of hydrogels and catalytic capabilities are in the majority of the cases the dynamic functionality of choice.

By summarizing and classifying the existing literature on multistrain ELMs, this review contributes to the development of innovations that boost a sustainable and circular economy. As these materials are solely made of or by biological cells, they could self-repair, biodegrade, or be derived from waste streams. More specifically, this review highlights that the use of multiple, specialized strains opens up a vast number of new capabilities and applications, among which the opportunity to design a fully biological material by combining a biological structural scaffold and a biological dynamic element. More control and predictability of multistrain interactions in general, could lead to a modular system, where an individual strain can be swapped for specific applications. Likewise, exploration of emerging behaviour in consortia can lead to new insights into ecological dynamics or community engineering. This in turn can lead to the discovery of new applications of consortia in material sciences. A multivalent metabolism is particularly interesting in complex substrates like wastewater or polluted soil. This is because both the functionality and viability of the consortium is boosted by respectively division of labour and cross-protection. This particular combination of features allows for adaptation to and functionality in extreme conditions. Multistrain bio-ELMs could this way revolutionize space or deep sea applications.

Whilst the research on both ELMs and microbial consortia is fast-growing, major bottlenecks, like social perception, biosafety, technical development, and regulation, prevent the development of industrially relevant multistrain ELMs thus far. Biosafety and technical development can be overcome by engineering strategies, which are merely a matter of time. Negative social perception and strict regulation, however, can prevent the distribution of a fully developed material, no matter how advanced or safe this material is. Therefore, education and exposure of the general public to living materials and synthetic biology are of utmost importance.

Altogether, this review article illustrates the revolutionary impact of (micro)biological consortia on the development of sustainable materials and shaping a more sustainable future for current and future generations. As well as providing insights into the diversity of living functionalities, the knowledge presented here can be utilized in a more rational approach to modular multistrain material engineering and gives a concise overview of the current research field that will boost further development of fully biological, multistrain ELMs.

A major take-home message is that this is an extremely promising and exponentially growing field, with a great amount of research opportunities as proven by the number of papers being published in the last couple of years. Combining the knowledge of (micro)biological and material disciplines has the potential to spark inventions that could transform both fields.
